# Durable response to FOLFOX plus serplulimab in AFP-producing gastric adenocarcinoma with liver metastases: a case report and literature review

**DOI:** 10.3389/fimmu.2026.1795732

**Published:** 2026-04-13

**Authors:** Yuqi Jin, Linglin Fu, Yuhan Zhao, Ziyan Tong, Yinuo Tan

**Affiliations:** 1 Department of Medical Oncology, Key Laboratory of Cancer Prevention and Intervention, Ministry of Education, The Second Affiliated Hospital, Zhejiang University School of Medicine, Hangzhou, Zhejiang, China; 2 The Second Affiliated Hospital, Zhejiang University School of Medicine, Hangzhou, China; 3 Cancer Center of Zhejiang University, Hangzhou, China; 4 Center for Medical Research and Innovation in Digestive System Tumors, Ministry of Education, Hangzhou, China; 5 Department of Infectious Diseases, The Second Affiliated Hospital, Zhejiang University School of Medicine, Hangzhou, China

**Keywords:** AFP-producing gastric carcinoma, alpha-fetoprotein, case report, FOLFOX, gastric cancer, hepatoid differentiation, liver metastasis, PD-1 blockade

## Abstract

**Background:**

AFP-producing gastric carcinoma (AFPGC) is an uncommon but clinically aggressive subset of gastric cancer with a strong propensity for liver metastasis. Serum alpha-fetoprotein (AFP) is often markedly elevated, whereas tumor AFP immunostaining can be negative, which may complicate recognition and classification.

**Case presentation:**

A 67-year-old man presented with epigastric pain. Contrast-enhanced computed tomography showed gastric wall thickening and multiple hepatic metastases. Endoscopic biopsy revealed a poorly differentiated adenocarcinoma with hepatoid/enteroblastic differentiation features. Immunohistochemistry was positive for SALL4, glypican-3, pan-cytokeratin, and nuclear CDX2, and negative for AFP and neuroendocrine markers. Helicobacter pylori testing was negative. Baseline serum AFP was markedly elevated. After intolerance to oral S-1, the patient received FOLFOX plus the PD-1 inhibitor serplulimab, followed by maintenance serplulimab. Treatment was well tolerated, without immune-related adverse events requiring systemic corticosteroids or treatment interruption.

**Outcomes:**

Serial magnetic resonance imaging demonstrated marked and sustained shrinkage of hepatic lesions. A RECIST v1.1–based assessment, using available measurements of measurable target lesions, was consistent with a partial response. Serum AFP rapidly normalized and remained within the reference range during maintenance therapy, paralleling the radiologic response.

**Conclusion:**

This case suggests that chemo-immunotherapy with platinum–fluoropyrimidine chemotherapy plus PD-1 blockade may yield substantial and durable disease control in selected patients with metastatic AFPGC, even when tumor AFP staining is negative. AFP kinetics provided a rapid and reproducible on-treatment biomarker that complemented imaging. Given the paucity of prospective data in AFP-high gastric cancer, this report is hypothesis-generating and supports further evaluation of chemo-immunotherapy in larger studies.

## Introduction

AFP-producing gastric carcinoma (AFPGC) is an uncommon but clinically important subset of gastric cancer characterized by markedly elevated serum alpha-fetoprotein (AFP) levels and an aggressive clinical course, with a strong propensity for hematogenous dissemination, particularly to the liver (1–3). AFPGC overlaps with gastric cancers showing hepatoid morphology; however, the term “gastric hepatoid adenocarcinoma” is traditionally reserved for tumors defined primarily by hepatoid histology and hepatocellular immunophenotypic features (4). In routine practice, AFP-high gastric cancers represent a heterogeneous spectrum that may exhibit hepatoid and/or enteroblastic differentiation features. Accordingly, strict classification as “hepatoid adenocarcinoma” can be challenging when hepatocellular markers are absent or when tumor AFP staining is negative (5). Therefore, diagnosis should rely on an integrated clinicopathologic approach that incorporates morphology, serum AFP levels, and an appropriate immunohistochemical (IHC) panel to distinguish AFP-high gastric cancer from primary hepatocellular carcinoma and from conventional gastric adenocarcinoma with focal hepatoid-like features.

Pathologically, AFPGC may show solid and trabecular growth patterns with abundant eosinophilic cytoplasm and pseudoacinar structures, resembling hepatoid differentiation (4). Tumors can express fetal-type gastrointestinal epithelial markers such as SALL4 and glypican-3, whereas neuroendocrine markers (synaptophysin and chromogranin A) are typically negative (4, 5). Cytokeratins and gastrointestinal-lineage markers, including pan-cytokeratin (CK-pan) and CDX2, can support a gastrointestinal origin (4). Importantly, tumor AFP expression is variable. Negative AFP staining in tumor tissue does not necessarily exclude an AFP-producing clinical phenotype when serum AFP is markedly elevated and the overall clinicopathologic correlation is concordant (6, 7).

Clinically, AFPGC is frequently diagnosed at an advanced stage and is often accompanied by synchronous liver metastases (8, 9). Outcomes are generally inferior to those of conventional gastric adenocarcinoma. Because of its rarity and biological heterogeneity, there is no unified disease-specific first-line standard for AFPGC (10). In current practice, systemic treatment is largely extrapolated from evidence in advanced gastric adenocarcinoma (11). For HER2-negative disease, platinum–fluoropyrimidine chemotherapy combined with PD-1 blockade has demonstrated overall survival benefits in multiple randomized trials and has been widely adopted as a first-line strategy (12–14). However, the applicability of chemo-immunotherapy to AFP-high gastric cancers remains insufficiently defined. These tumors are underrepresented in prospective trials, and the available evidence is dominated by retrospective series and case reports (15).

Against this background, we report a patient with metastatic AFPGC with hepatoid/enteroblastic differentiation features who achieved a radiologic partial response and durable disease control with FOLFOX plus the PD-1 inhibitor serplulimab, followed by maintenance serplulimab. The rapid decline and normalization of serum AFP paralleled radiologic tumor shrinkage. We describe the clinicopathologic findings, treatment course, response assessment, and follow-up. We also discuss this case in the context of the emerging literature on immunotherapy-based combinations in AFP-high gastric cancer.

## Case presentation

### Patient information

A 67-year-old man presented with a 6-month history of recurrent epigastric pain. On March 26, 2024, contrast-enhanced abdominal computed tomography (CT) performed at an outside hospital demonstrated diffuse gastric wall thickening, enlarged regional lymph nodes, and multiple mildly hypodense hepatic lesions, raising suspicion for metastatic gastric cancer. He was subsequently referred to our center for further evaluation.

Esophagogastroduodenoscopy performed on March 29, 2024, revealed multiple gastric ulcers, one of which was highly suspicious for malignancy. On April 2, 2024, biopsy specimens obtained from the anterior wall of the lower gastric body showed ulcerated mucosa infiltrated by a poorly differentiated carcinoma arranged predominantly in solid and trabecular nests with focal pseudoacinar structures. The tumor cells were large and polygonal, with abundant eosinophilic cytoplasm, centrally located nuclei, and conspicuous nucleoli, displaying morphologic features suggestive of hepatoid/enteroblastic differentiation.

**Figure 1:**
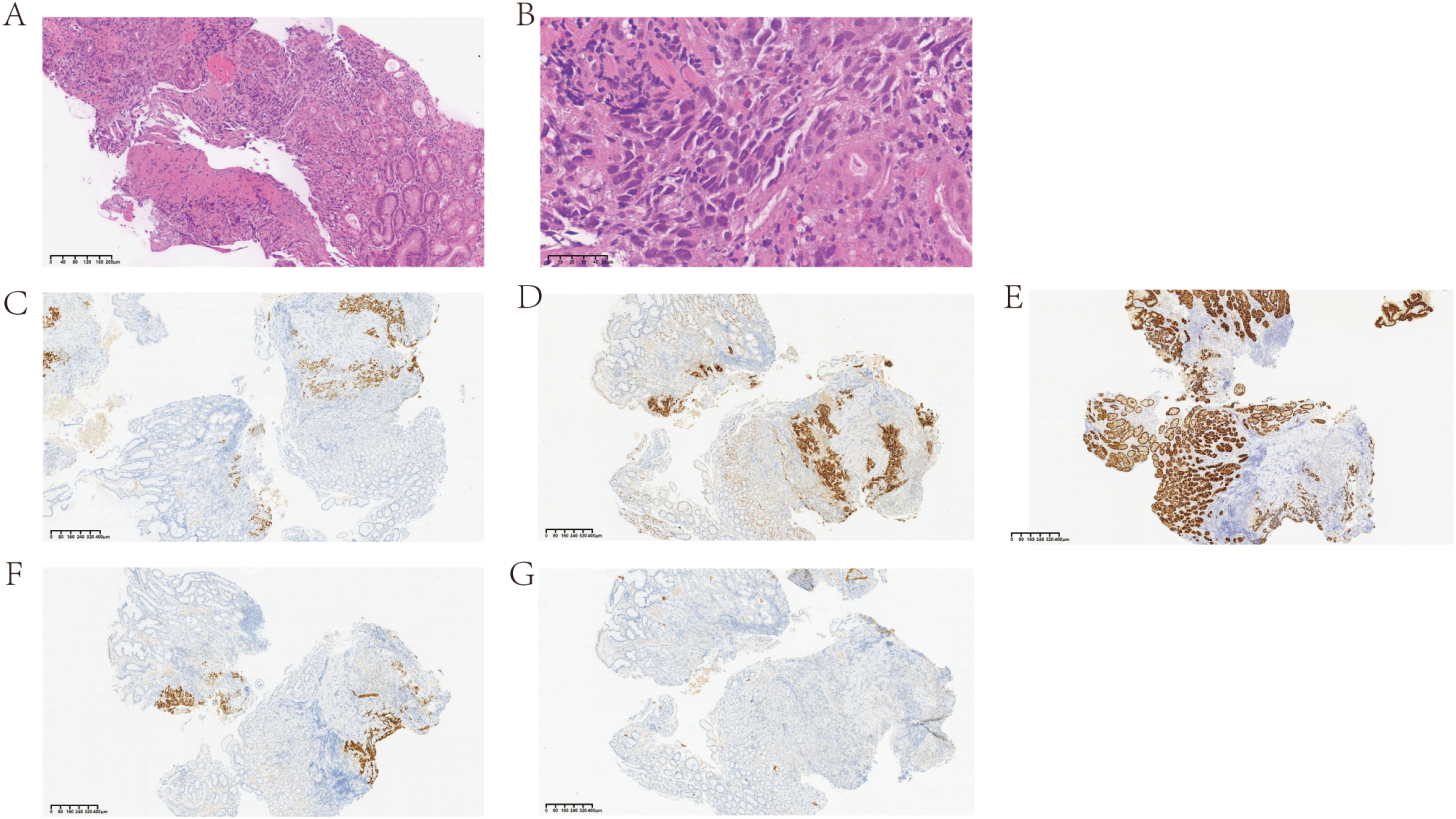
Gastric hepatoid adenocarcinoma—histology and immunophenotype. **(A)** H&E, ×100: ulcerated gastric mucosa with solid/trabecular tumour nests. **(B)** H&E, ×400: polygonal tumour cells with abundant eosinophilic cytoplasm and prominent nucleoli. **(C)** SALL4, nuclear-positive in tumour cells. **(D)** Glypican-3, cytoplasmic/membranous-positive. **(E)** Pan-cytokeratin, positive **(F)** CDX2, nuclear-positive, supporting gastrointestinal origin. **(G)** AFP immunostaining negative in tumour cells despite markedly elevated baseline serum AFP.

Immunohistochemistry (IHC) demonstrated diffuse positivity for pan-cytokeratin (CK-pan) and nuclear CDX2, together with strong expression of SALL4 and glypican-3 (GPC3), supporting a fetal-type gastrointestinal epithelial phenotype. AFP staining was negative in the tumor cells, and synaptophysin and chromogranin A were also negative. Hepatocellular markers, including HepPar-1, were negative, arguing against primary hepatocellular carcinoma in the setting of multifocal hepatic lesions. The histopathological and immunohistochemical findings are shown in **Figure 1**. All four mismatch-repair proteins (MLH1, PMS2, MSH2, and MSH6) showed retained nuclear expression, consistent with an MMR-proficient tumor. Helicobacter pylori testing was negative.

At our institution, baseline laboratory tests obtained on April 8, 2024, revealed a markedly elevated serum AFP level of 1, 131.3 ng/mL (reference range, <20 ng/mL), despite negative AFP staining in tumor tissue. Taken together, the presence of a primary gastric carcinoma with hepatoid/enteroblastic morphology, a fetal-type immunophenotype (SALL4+/GPC3+/CDX2+), negative hepatocellular-lineage markers (HepPar-1−), and synchronous multifocal liver metastases supported classification of the tumor as AFP-producing gastric carcinoma (AFPGC) with hepatoid/enteroblastic differentiation features, rather than a strict diagnosis of gastric hepatoid adenocarcinoma or primary hepatocellular carcinoma.

### Diagnostic and therapeutic course

Staging abdominal CT and liver magnetic resonance imaging (MRI), performed on April 8, 2024, confirmed a primary gastric mass with bulky regional lymphadenopathy, multiple liver metastases, portal vein tumor thrombus, omental deposits, and ascites, consistent with cT3N2M1 disease according to the 2022 CSCO guidelines for gastric cancer. Given the extensive hepatic involvement and poor surgical candidacy, systemic therapy was selected after multidisciplinary discussion.

On April 10, 2024, the patient received first-line chemotherapy with oxaliplatin 220 mg combined with an S-1–based regimen, along with standard antiemetic, gastroprotective, and hydration support. Because of intolerance to oral S-1 during the first cycle, the regimen was modified from the second cycle onward to FOLFOX plus serplulimab every 2 weeks, consisting of oxaliplatin 150 mg, leucovorin 0.7 g, 5-fluorouracil 0.7 g as a bolus followed by 4 g continuous infusion, and serplulimab 200 mg administered intravenously on day 1 of each 14-day cycle.

**Figure 2:**
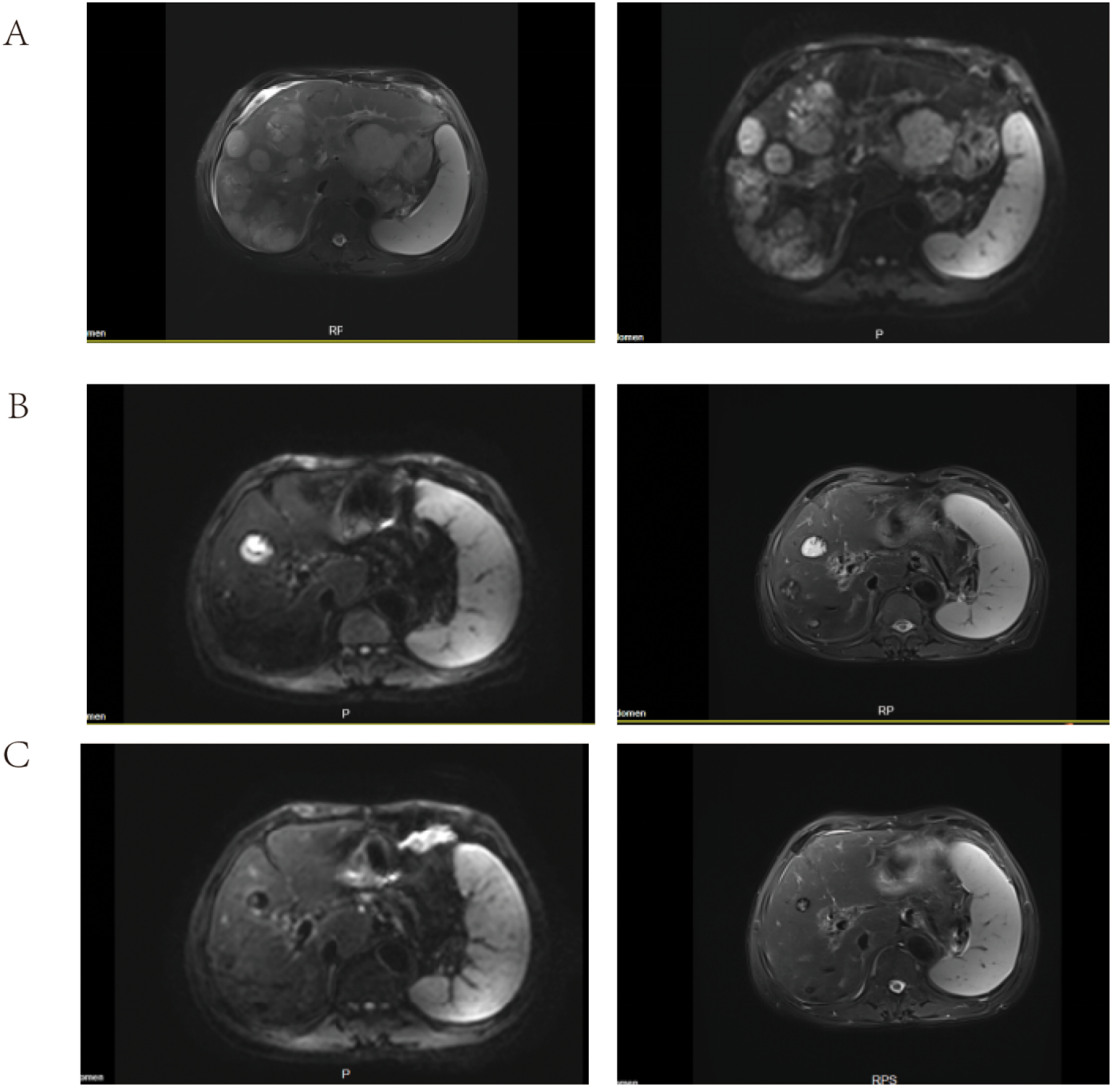
Liver MRI demonstrating treatment response over time. **(A)** April 7, 2024 (baseline) shows multiple hepatic metastases. **(B)** June 7, 2024, during FOLFOX plus serplulimab, shows interval shrinkage of target lesions. **(C)** Sept 16, 2024 shows continued reduction without new lesions.

The patient continued combination therapy until November 12, 2024, when cytotoxic chemotherapy was completed, for a total of 15 cycles of FOLFOX plus serplulimab. Interval CT and MRI scans demonstrated progressive shrinkage of the hepatic and nodal metastases compared with baseline, meeting the criteria for partial response (PR) according to RECIST version 1.1 (**Figures 2A–C**). No dose reductions of oxaliplatin or 5-fluorouracil were required, and there were no treatment delays longer than 1 week.

### Maintenance therapy and follow-up outcomes

**Figure 3:**
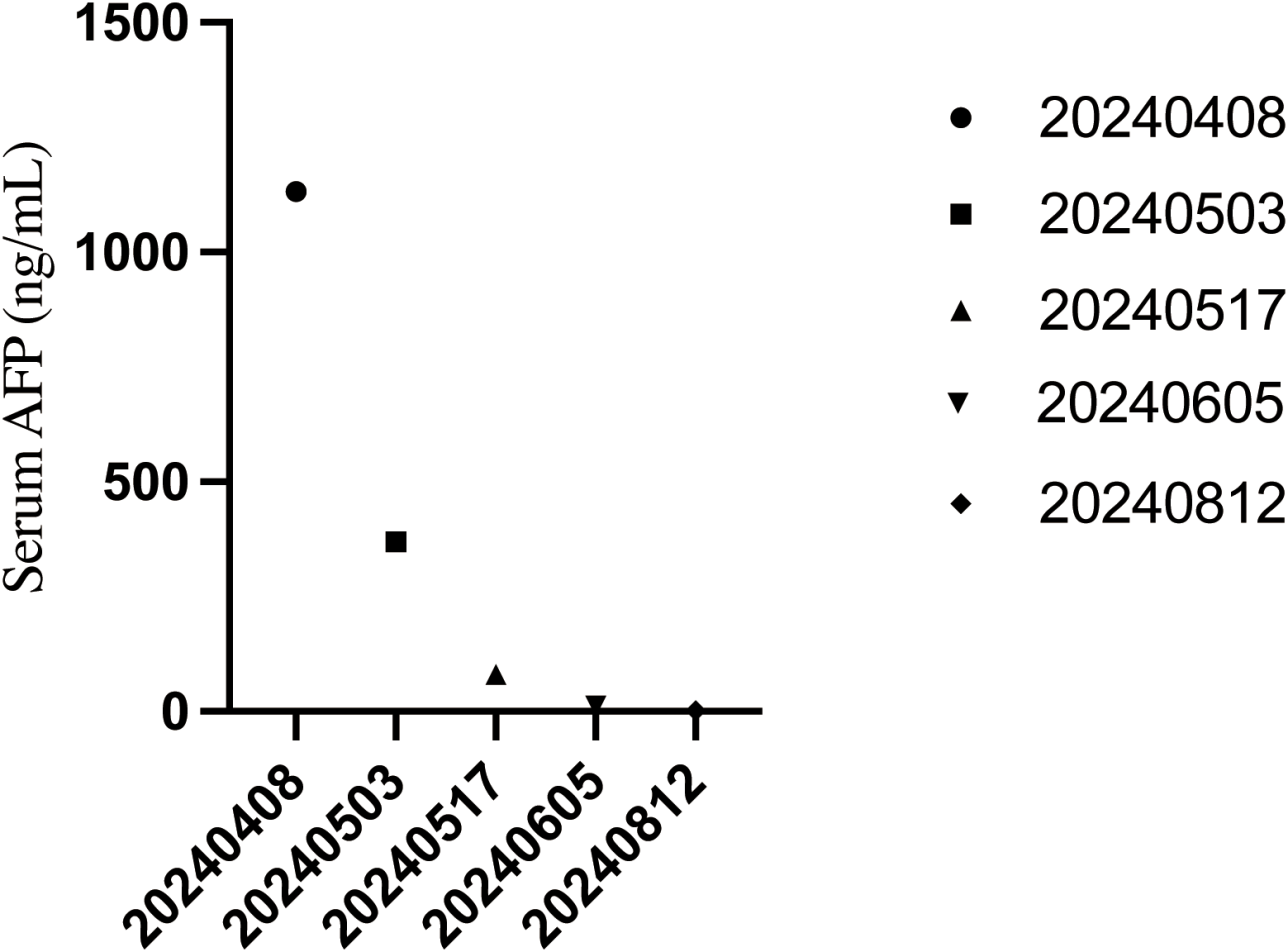
On-treatment AFP kinetics.Serum alpha-fetoprotein (AFP) levels declined from 1, 131.3 ng/mL at baseline on 8 April 2024 to 369.9 ng/mL on 3 May, 81.2 ng/mL on 17 May, 12.2 ng/mL on 5 June, and 1.8 ng/mL on 12 August 2024. AFP entered and remained within the normal reference range (< 20 ng/mL) within four months of treatment initiation.

**Figure 4:**
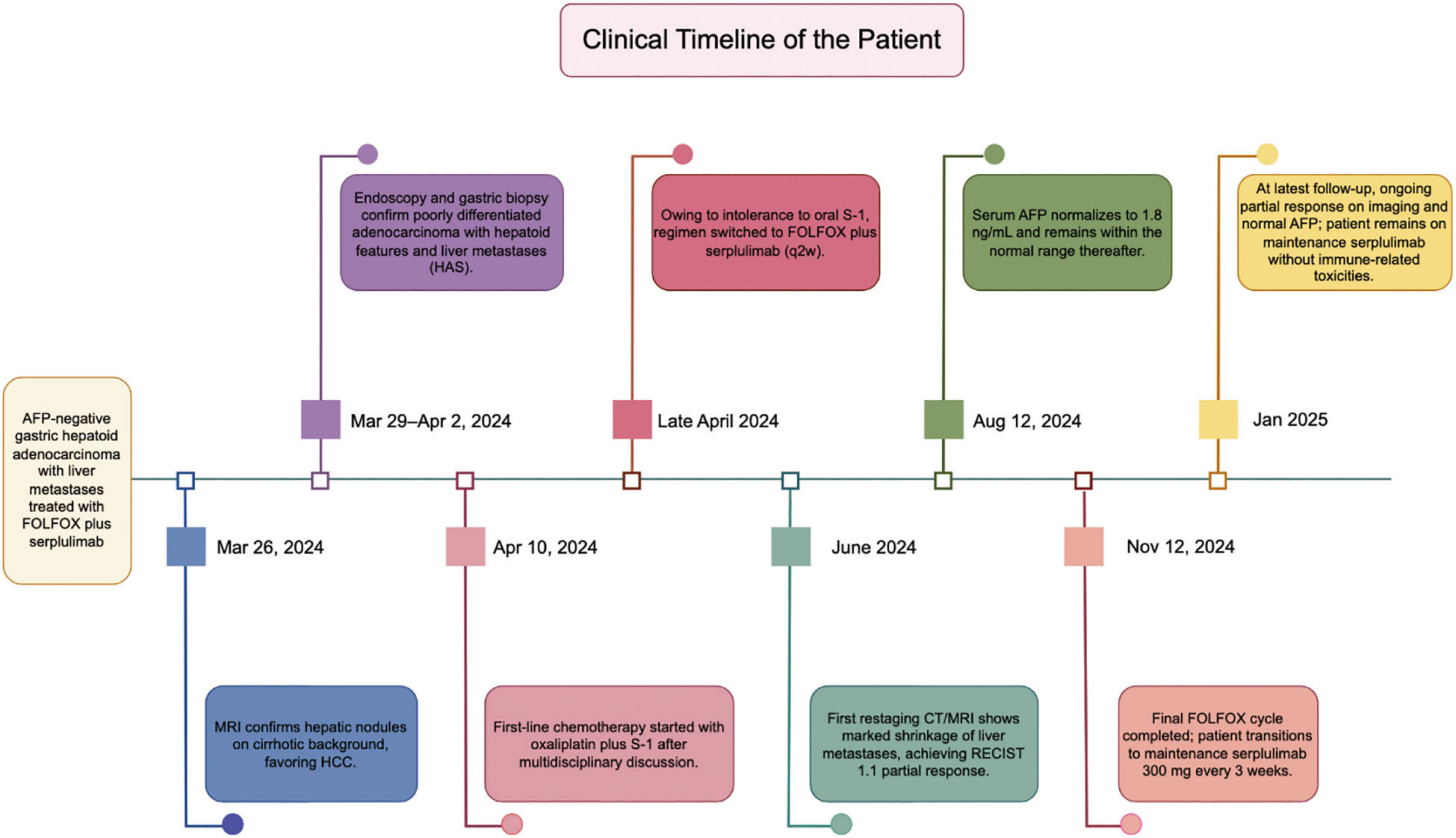
Diagnostic and therapeutic timeline of the patient.

During maintenance therapy, serial imaging demonstrated further reduction or sustained stabilization of the lesions, without evidence of new disease. The patient’s epigastric pain improved markedly. At the most recent follow-up, he remained in PR and continued maintenance serplulimab with close surveillance. Serum AFP returned to the normal range and remained suppressed during follow-up (**Figure 3**). A detailed clinical timeline summarizing the diagnostic procedures, systemic treatment, imaging assessments, and AFP measurements is provided in **Figure 4** (created by figdraw).

### Literature review

To contextualize the role of PD-1–based immunotherapy in AFP-high gastric cancer, we summarized selected clinically relevant studies, including pivotal first-line trials in advanced gastric cancer, reports involving AFP-producing gastric cancer, and a serplulimab-based study in hepatic malignancy, as shown in **Table 1**.

**Table 1:** Selected evidence of PD-1–based immunotherapy in advanced gastric cancer and AFP-high subsets.

No.	Year	Journal	Study type	Tumor type/population	Immunotherapy regimen	Key findings	DOI
1	2021	Lancet	Phase III randomized, open-label trial (CheckMate 649)	Previously untreated, unresectable, non-HER2-positive gastric, gastro-oesophageal junction, or oesophageal adenocarcinoma	Nivolumab + chemotherapy vs chemotherapy alone	Nivolumab plus chemotherapy improved OS and PFS versus chemotherapy alone and became a landmark first-line PD-1–based regimen in advanced gastric/GEJ adenocarcinoma.	10.1016/S0140-6736(21)00797-2
2	2023	Lancet Oncology	Phase III randomized, double-blind trial (KEYNOTE-859)	HER2-negative advanced gastric or gastroesophageal junction adenocarcinoma	Pembrolizumab + chemotherapy vs placebo + chemotherapy	Pembrolizumab plus chemotherapy significantly improved overall survival with manageable safety, supporting first-line PD-1 blockade in HER2-negative advanced gastric cancer.	10.1016/S1470-2045(23)00515-6
3	2023	JAMA	Phase III randomized clinical trial (ORIENT-16)	Unresectable locally advanced, recurrent, or metastatic gastric or gastroesophageal junction adenocarcinoma	Sintilimab + chemotherapy vs placebo + chemotherapy	Sintilimab significantly improved overall survival in all randomized patients and in the CPS ≥5 subgroup.	10.1001/jama.2023.19918
4	2020	Cancer Management and Research	Retrospective clinical study	Advanced gastric cancer patients with elevated serum AFP or hepatoid adenocarcinoma of the stomach	PD-1 inhibitor (nivolumab) + chemotherapy vs non-ICI systemic therapy	In 21 patients, the immunotherapy group had mPFS 22.0 months versus 4.3 months in controls; mOS was not reached in the immunotherapy group, suggesting potential benefit of chemo-immunotherapy in AFP-high/HAS populations.	10.2147/CMAR.S276969
5	2024	Frontiers in Immunology	Case report	AFP-producing gastric cancer	Tislelizumab + SOX chemotherapy	Reported a significant response of AFPGC to combined chemotherapy and immunotherapy, supporting the possible activity of PD-1–based combinations in this rare subtype.	10.3389/fimmu.2024.1448875
6	2025	Cancer Immunology, Immunotherapy	Open-label, multicenter phase II study	Advanced hepatocellular carcinoma	Serplulimab ± bevacizumab biosimilar HLX04; first-line cohort received serplulimab + HLX04	The study reported manageable safety and preliminary efficacy of serplulimab-based therapy in advanced HCC, providing hepatic-disease context for PD-1–based treatment feasibility.	10.1007/s00262-024-03917-w

## Discussion

AFP-producing gastric carcinoma (AFPGC) is a biologically aggressive subtype of gastric cancer that is frequently associated with hematogenous dissemination, particularly to the liver, and generally poor outcomes compared with conventional gastric adenocarcinoma (16, 17). In this context, our patient, who was HER2-negative and mismatch-repair proficient (pMMR), presented with multifocal liver metastases and a markedly elevated serum alpha-fetoprotein (AFP) level despite negative AFP staining in tumor tissue (18). He achieved a durable partial response (RECIST v1.1) to oxaliplatin/fluoropyrimidine chemotherapy combined with the PD-1 inhibitor serplulimab, followed by maintenance serplulimab (19). Notably, the rapid normalization of serum AFP paralleled radiologic tumor shrinkage and sustained symptom relief, suggesting possible activity of chemo-immunotherapy in this metastatic AFPGC case and illustrating the potential utility of AFP kinetics as an exploratory on-treatment biomarker (18, 20).

Serplulimab (HLX10) is a humanized IgG4 anti-PD-1 antibody supported by randomized evidence for its addition to chemotherapy in other malignancies (21). In extensive-stage small-cell lung cancer, the phase 3 ASTRUM-005 trial demonstrated an overall survival benefit for serplulimab plus etoposide–platinum with manageable safety (22). Signals of activity in hepatic disease have also been reported in hepatocellular carcinoma when serplulimab was combined with anti-angiogenic therapy (23). Although these data are not derived from gastric cancer trials, they provide biological plausibility for PD-1–based combinations in patients with extensive liver involvement and support the feasibility of serplulimab-containing regimens from a tolerability perspective. Together with the durable tumor control and AFP normalization observed in our patient, these observations provide a hypothesis-generating rationale for exploring serplulimab-based chemo-immunotherapy in AFPGC in future disease-specific studies.

Although randomized evidence has established chemotherapy plus PD-1 blockade as a first-line standard for advanced HER2-negative gastric cancer, its applicability to AFPGC remains uncertain because AFPGC is biologically distinct and underrepresented in prospective trials (24). Retrospective series and case-based reports suggest heterogeneity in immunotherapy sensitivity among AFP-high gastric cancers, and some observations indicate that immunotherapy alone may be insufficient in a subset of patients (25, 26). Mechanistically, AFPGC with hepatoid/enteroblastic differentiation features has been associated with an “immunologically cold” tumor microenvironment, including low tumor mutational burden, microsatellite stability, limited cytotoxic T-cell infiltration, and enrichment of immunosuppressive myeloid and regulatory T-cell populations, often accompanied by low PD-L1 expression (24). In addition, aberrant angiogenesis and hypoxia may further constrain immune-cell trafficking and effector function (27, 28). AFP itself has been implicated in immunosuppressive and pro-angiogenic signaling in experimental and clinical settings, providing a plausible link between AFP elevation, aggressive behavior, and reduced responsiveness to immunotherapy monotherapy (1, 29–31). Within such a microenvironment, cytotoxic chemotherapy may act as an immunologic primer by enhancing antigen release and presentation, inducing immunogenic cell death, and transiently depleting suppressive immune subsets, thereby facilitating synergy with PD-1 inhibition. The depth and durability of response in this single case are consistent with this immunologic model, but whether chemo-immunotherapy is superior to immunotherapy alone in metastatic AFPGC requires validation in larger cohorts (32).

The diagnostic features of this case also warrant emphasis, particularly because terminology and strict criteria for “hepatoid adenocarcinoma” remain debated (4). Early recognition of AFP-high gastric cancer can be challenging because clinical manifestations and imaging findings are nonspecific, and extensive liver involvement may prompt consideration of primary hepatocellular carcinoma. Therefore, an integrated clinicopathologic approach is essential. In our case, the tumor showed a trabecular/solid growth pattern with abundant eosinophilic cytoplasm and focal pseudoacinar structures, together with strong SALL4 and glypican-3 expression and nuclear CDX2 positivity, supporting a fetal-type gastrointestinal epithelial phenotype with hepatoid/enteroblastic differentiation features (7, 29). Importantly, both tissue AFP staining and HepPar-1 were negative, making a strict designation of “gastric hepatoid adenocarcinoma” debatable if hepatocellular-lineage markers are required. However, negative tissue AFP staining has been reported in AFP-high gastric cancers and should not preclude classification as AFPGC when serum AFP is markedly elevated and the overall clinicopathologic correlation is concordant. Accordingly, we consider this case best classified as AFPGC with hepatoid/enteroblastic differentiation features, an approach that is diagnostically conservative yet biologically aligned with the clinical phenotype and biomarker behavior.

An important diagnostic challenge in this case was the distinction among AFP-producing gastric carcinoma, hepatoid adenocarcinoma of the stomach, and hepatocellular carcinoma, particularly in the setting of markedly elevated serum AFP and multiple liver lesions. Hepatoid adenocarcinoma of the stomach is a well-recognized differential diagnosis because it can share morphologic and serologic features with hepatocellular carcinoma and shows a marked propensity for liver metastasis (33). In the present case, the diagnosis was supported by integrated clinicopathologic findings, including endoscopic evidence of a gastric primary tumor, histopathologic confirmation of poorly differentiated adenocarcinoma, and the immunophenotypic profile with SALL4 and glypican-3 expression, markers that are useful in identifying fetal gut–type differentiation and in separating gastric hepatoid/enteroblastic neoplasms from primary hepatocellular carcinoma (34). Notably, negative AFP immunohistochemistry does not necessarily exclude this spectrum of tumors, as gastric adenocarcinoma with enteroblastic differentiation may retain SALL4/glypican-3 expression with absent or only limited AFP staining (34, 35). Taken together, the overall findings were considered more consistent with AFP-producing gastric carcinoma with hepatoid/enteroblastic differentiation features and liver metastases than with primary hepatocellular carcinoma.

Serum AFP remains one of the most clinically useful biomarkers in AFPGC. Beyond its diagnostic value, AFP may serve as a dynamic indicator of treatment response during systemic therapy. In our patient, AFP declined rapidly from a markedly elevated level to the normal range, paralleled radiologic tumor regression, and remained suppressed during maintenance immunotherapy (20, 36). This concordance supports routine AFP monitoring as an adjunct to imaging for on-treatment assessment and potentially for early detection of disease progression. Prospective studies are needed to define the AFP thresholds and kinetic patterns that best predict durable benefit or impending progression under chemo-immunotherapy.

Although limited to a single case, our report may offer preliminary clinical insights.For patients with metastatic, HER2-negative, pMMR/microsatellite-stable AFPGC, a platinum/fluoropyrimidine backbone combined with PD-1 inhibition appeared feasible in this patient and was associated with meaningful tumor control; however, generalizability remains uncertain (37). When initial responses are suboptimal or resistance emerges, strategies that remodel the tumor microenvironment—such as approaches that improve perfusion or alleviate hypoxia—may represent rational avenues to enhance immunotherapy efficacy, given the roles of angiogenesis and oxygenation in shaping antitumor immunity in AFP-high gastric cancers. These hypotheses merit testing in translationally enriched, biomarker-driven studies that incorporate serial immune profiling alongside AFP dynamics.

In summary, chemo-immunotherapy in this patient with metastatic AFPGC was associated with substantial and sustained clinical benefit accompanied by AFP normalization. While encouraging, this observation from a single patient is hypothesis-generating and should be validated in larger studies to clarify which AFPGC subgroups derive the greatest benefit and to establish biomarker strategies that can reliably guide treatment. Given the absence of disease-specific prospective data in AFPGC, these findings should not be interpreted as establishing a standard of care.

## Data Availability

The original contributions presented in the study are included in the article/supplementary material. Further inquiries can be directed to the corresponding author.
